# Person-centered and youth-oriented interventions to improve TB Care for adolescents and young adults

**DOI:** 10.1371/journal.pgph.0003659

**Published:** 2024-11-15

**Authors:** Patricia Waterous, Rudo Chingono, Constance Mackworth-Young, Chipo Nyamayaro, Faith Rutendo Kandiye, Edson Marambire, Joanna Schellenberg, Junior Mutsvangwa, Prosper Chonzi, Fungai Kavenga, Rashida Ferrand, Katharina Kranzer, Sarah Bernays

**Affiliations:** 1 Department of Clinical Research, London School of Hygiene and Tropical Medicine, London, United Kingdom; 2 Biomedical Research and Training Institute, Harare, Zimbabwe; 3 Department of Global Health and Development, London School of Hygiene and Tropical Medicine, London, United Kingdom; 4 Department of Disease Control, London School of Hygiene and Tropical Medicine, London, United Kingdom; 5 Harare City Health, Harare, Zimbabwe; 6 National Tuberculosis Program, Harare, Zimbabwe; 7 Division of Infectious Diseases and Tropical Medicine, LMU University Hospital, LMU Munich, Munich, Germany; 8 School of Public Health, University of Sydney, Sydney, Australia; National University of Singapore, SINGAPORE

## Abstract

**Introduction:**

Globally adolescents and young adults (AYA) with tuberculosis (TB) face unique challenges. Until recently they have received little attention and few tailored interventions exist. To improve TB outcomes in this population, there is a need to implement tailored interventions. However, limited research has been conducted about how to meet the needs of AYA with tuberculosis. In this paper we present the findings of a qualitative study to explore the needs of AYA with TB in Zimbabwe and to identify interventions to optimize their engagement in TB care.

**Methods:**

We conducted two participatory workshops with 16 AYA, aged 10–24 years diagnosed with TB to explore their experiences of TB disease and treatment. Through subsequent interviews with 15 of the same AYA and with two other key stakeholder groups (health care providers n = 11 and policy makers n = 9), we sought to identify areas of convergence and divergence about what youth-orientated services and policies would be effective in Zimbabwe. Qualitative data were analyzed iteratively and thematically.

**Results:**

The findings are presented to align with four levels of a socio-ecological framework: individual, community, health system and policy. All three stakeholder groups highlighted the unmet mental health and TB literacy needs of AYA, which are particularly acute early in their TB care journey, as well as the imperative of engendering family support and securing the continuity of educational or employment opportunities during and after receiving TB care. There was a consensus that clinical services needed to become more youth-centered by extending training for health care providers and investing in peer-delivered psychosocial support. More broadly, there was also a strong consensus that adolescent-specific TB policies require further development and implementation, accompanied by community-based TB education and awareness campaigns to emphasize the curability of TB and to reduce TB related stigma.

**Conclusions:**

There is much to be done to improve TB care for AYA. We found that there is need for alignment on where investment is needed to support the development of context-appropriate and effective interventions. There is an opportunity to benefit from translational learning from other successful approaches, such as HIV, within the region. Implementation of evidence-based interventions and youth-friendly policies and programs are much needed to improve outcomes for AYA with TB.

## Introduction

Tuberculosis (TB) is the leading infectious disease killer in the world with an estimated 10.6 million people having developed TB in 2022 and 1.3 million people died from TB [1]. Until recently, the TB community has mainly focused on young children and adults. This is despite the fact that an estimated 1.8 million adolescents (aged 10–19) and young adults (aged 19–24) develop TB each year [2]. Diagnostic delays in adolescents and young adults (AYA) are common [3–5],suboptimal adherence has been reported [6, 7] and AYA thus have poorer TB outcomes than other age groups [8]. TB risk increases substantially post-puberty and onward transmission of *Mycobacterium tuberculosis* (Mtb) from AYA is more likely compared to young children [9–11]. This has been exacerbated by limited testing within primary healthcare for this age group [12, 13] due to a lack of awareness that TB affects young people.

Globally, there is a call for AYA with TB to receive particular attention [14–16]. This is reflected in the recent World Health Organization (WHO) 2022 updated guidelines [17] which recommends the adoption of a more youth-centred approach (those that are equitable, accessible, acceptable, appropriate and effective [18]) to better meet the needs of AYA in the delivery of TB care. However, action to redress the challenges that AYA living with TB face lags behind. This is in part due to the limited evidence base about how to transform and improve existing services for AYA within the resource-scarce health systems that tend to characterise high-TB prevalence settings.

Learning from research focused on AYA living with HIV or diabetes [19–23] illustrates the complexity of sustaining treatment engagement during the developmental phase of adolescence because of how it collides with conflicting social, relational and educational priorities in their lives. Despite the opportunities presented by this translational knowledge, there remains a need to understand the particularities of the challenges and needs provoked by living with TB, a clinically and socially complex, but curable, infectious disease.

We conducted a qualitative study in Zimbabwe to explore with AYA living with TB themselves and key stakeholders responsible for the design and delivery of TB care (healthcare providers and policy makers) what TB treatment challenges are experienced and what interventions are needed to support the delivery of AYA TB care which can lead to improved related outcomes, including experiences and well-being.

## Methods

This qualitative study was conducted in Harare, Zimbabwe with recruitment taking place 04/03/2021 through 31/10/2021. At the time of the study Zimbabwe was a high TB-burden country [24] with an estimated TB incidence of 199/100,000 [25] and an HIV prevalence of 11.8% in 15–45 year olds [26]. In Zimbabwe TB treatment is free of charge. Yet, the major drivers of catastrophic costs lay outside the healthcare sector such as income loss due to loss of productivity time, travel costs and nutritional supplements [27]. A policy regarding isolation requirements does not currently exist in Zimbabwe, yet most healthcare providers advise patients to self-isolate for at least 2 weeks following their diagnosis.

### Study population

Sixteen AYA living with TB, aged 16–24, and having received at least 2 weeks of TB treatment were recruited to participate in the study. Purposive sampling was used in recruiting participants to represent the entire age range and array of length of times on treatment. Recruitment was based on a target sample size of 10–20 interviews and reaching saturation. These AYA participants alongside healthcare providers and policy stakeholders were all interviewed between 01/04/2021 and 31/10/2021. Interviews with AYAs were conducted at one of the seven primary healthcare clinics (PHCs) in the high-density suburbs of Harare in Zimbabwe. These PHCs provide nurse-led, acute primary care for the treatment of locally common infections, including the diagnosis of and treatment for HIV and TB. Individuals are assigned to the PHC closest to their home where operating hours are weekdays from 8am to 5pm. They were selected as recruitment sites because of their high number of TB notifications across all age groups. Individuals diagnosed with TB, regardless of age, usually visit the clinic for drug refills and treatment monitoring once a week during the intensive phase of treatment, which ordinarily lasts for two months, and then again once every two weeks during the four-month continuation phase.

Eligible AYA were identified by polyclinic staff using the National Tuberculosis Programme (NTP) registers and clinic attendance. They were invited to participate by polyclinic nurses and there were no refusals. Written informed consent was provided by all AYA over the age of 18 and consent from parents or guardians were provided for AYA between the ages of 16–18. The participants ranged in age from 16 to 24 years, with the majority of participants (13/16) aged between 18 and 24 years. There was an equal number of male (n = 8) and female (n = 8) participants. All had been on TB treatment between two to seven months at the time of data collection. Twelve participants were HIV negative and four were living with HIV.

Eleven healthcare providers, who were working directly with AYA affected by TB in their primary roles (i.e. TB nurse) at the seven PHCs, were invited and provided written informed consent. They were selected based on the recommendations of the head nurse/matron of each clinic. Nine policy stakeholders were invited to participate and provided written informed consent. They were identified using purposive sampling to ensure that key organizations influencing relevant policies were represented within the study. They were recruited from the National Tuberculosis Programme, the AIDS and TB unit within the Ministry of Health as well as district officers who are employees of the Ministry of Health tasked to oversee the implementation of specific activities within the district, including the PHCs. In addition some individuals were recruited from Harare City Health, a municipality health directorate, as well as country/regional WHO staff.

### Data collection

The sixteen AYA participated in a four-hour participatory workshop facilitated in Shona by two experienced Zimbabwean qualitative researchers (CN and FRK). Two workshops were held with groups of nine and seven participants, based on AYA availability for each scheduled workshop. None of the AYA participants were previously known to one another. Each workshop was conducted by the study researchers in the local language of Shona within the compound of a recruitment polyclinic. Participatory activities including collages [28], buzz groups and brainstorms were used in the workshops to generate collective narratives (i.e. shared individual experiences) through group interactions. An example of how this worked is that participants were provided a set of exploratory questions to discuss in smaller groups (or 2–3 each) to encourage active participation, discussion and sharing of experiences before coming together as a larger group to discuss further. Topics covered included the AYA TB journey, its related impact on their wellbeing, and service provision for this age group.

Fifteen of these AYA then participated in individual in-depth interviews a few weeks later, with one AYA declining. These in-person interviews, assisted with semi-structured guides, were conducted in Shona by one of two experienced Zimbabwean qualitative researchers (CN and FRK). They were held at one of the PHCs and lasted 30–45 minutes. Interviews with AYA focused on their individual TB journeys to date, including service provision, psychosocial challenges and treatment adherence. This gave an opportunity to explore more personal experiences, which may not have been adequately explored in the workshops given the method’s focus on collective narratives.

The interviews with healthcare providers explored barriers to TB diagnosis and treatment, adolescent well-being and AYA medical care using semi-structured guides and were conducted in Shona or English by experienced qualitative researchers (CN, FRK and PW), at polyclinics. Interviews with policymakers were conducted in English by PW. Interviews with both stakeholder groups lasted between 30–45 minutes. Data were audio recorded and transcribed and once validated the audio-files were deleted. Shona transcripts were translated verbatim into English. Pseudonyms were used in transcripts and in this manuscript to protect participant anonymity. Fieldnotes and photos of workshop materials was included in the analysis.

### Data analysis

Familiarization codes were identified deductively and inductively after reading the workshop and in-depth interview transcripts, and a coding framework was developed. The in-depth inductive thematic analysis consisted of six steps including: data familiarization, keyword identification, code selection, theme development, conceptualization through the interpretation of keywords, codes and themes, and finally the development of a conceptual model [29]. The deductive coding, informed by previous work in this area [30–32], drew on the four levels conceptualized within the socioecological framework: i) individuals, ii) family/community, iii) provider/health facility and iv) policy and health system. The analysis of the three datasets (AYA, healthcare providers and policymakers) was initially conducted separately and then these analyses were triangulated to identify areas of similarity and divergence. Data were organized and coded using NVivo version 12. A constant-comparative approach was adopted to support the thematic analysis, utilizing elements of grounded theory where excerpts of raw data are sorted according to attributes and organized in a structured way to formulate a theory [29]. Analytical ideas identified through the coding process were discussed in frequent team discussions (CMY, SB and PW) and subsequently developed further through analytical memos. The analysis is presented through three indicative case studies, in which we draw together the data collected with each participant to explain their treatment journey and experience. These participants went through the same data collection process as all the other participants, as described above, but were selected due to the commonality of their experiences with others in the sample and the richness of their accounts.

### Ethical approval

Ethical approvals were granted by the ethics committees of London School of Hygiene and Tropical Medicine (22764) and the Biomedical Research and Training Institute in Zimbabwe Institutional Review Board: IRB (AP154/2019), the Medical Research Council of Zimbabwe (MRCZ/A/2569. Written informed consent was collected prior to participation. For the AYA participants aged under 18, written consent was sought first from their guardian, followed by their assent. No incentives were provided to participants. However, participants received transport reimbursement and refreshments during the full day workshops.

## Results

### Needs of AYA with TB

All AYA participants described having unmet acute needs. Although there was some variation across the sample in their specific circumstances, we identified consistent features of their experiences which indicated commonalities in the challenges that they faced. To demonstrate this, we present three case studies, which reflect the dominant pattern identified within the data. These case studies illustrate the AYA participants’ key unmet needs, as well as highlight the disruptive implications this had for their wellbeing and engagement with TB care. We first present three case studies illuminating AYA needs according to the socio-ecological domains (levels) and further expanded in Table 1. In the second part of the results, we describe the interventions which participants suggested could respond to these needs.

**Table 1:** AYA TB challenges with indicative insights from adolescents and young adults, healthcare provider and policy makers, according to socio-ecological framework.

Major theme	Adolescents and young adults	Healthcare provider		Policy maker
Domain				Individual	
**Financial issues**	“There is a lot of money that is needed…the tests that were needed to be done to confirm the illness…I think I had to do three tests and they would cost $20 USD and up…Tests for blood, for sputum, for x-ray….” [Tafadzwa, male, aged 23] “So, money became a problem for food, my sister lost her job because they were saying she was missing too many days of work” [Chipo, female, aged 18]	“Financial issues to buy food and even sometimes for transport to get to the clinic.” [#4, female] “Yes, there is a difference because some patients have difficulty finding food to eat while they are taking their drugs” [#8, female]		“So catastrophic costs for TB are a major issue…a patient will suffer lots and lots of costs just to go for TB diagnosis, these costs are very high for our patients and they are very prohibitive.” [#3, male, national] “Food insecurity is also a big issue for some of our districts where they are unable to get the right nutrition which may also be contributing to TB.” [#1, male, national]
**Disrupted education**	“It would be difficult for me to balance my time for school and time to collect pills” [Tafadzwa, male, aged 23] “I did not go to school for about 3 months” [Chipo, female, aged 18]	“I think to make it easier they should not be forced to come in-person to the clinic too often. Because some of them are college students and some of them are going to school so they are bearing too much on their own.” [#8, female] “Yes because most of the time they will miss their lessons coming here to collect their drugs.” [#7, female] “An adolescent with DR-TB, the doctor also had to write a letter to the [university] manager so that she would be allowed to stay in her own room [to isolate]- since mostly in these universities they are sharing [rooms].” [#6, female]		“The big one is education—how to be able to continue through schooling if that’s what they’re doing.” [#8, male, international]
**Psychosocial and mental health challenges**	“I was told that people get infected with TB because of stress for long periods or from drinking alcohol and smoking and I was doing that” [Garikai, male, aged 22] “When I first got sick I did not have anyone to look after me–people would actually say that one day I will wake up dead” [Fadzai, female, aged 19] “I was going through a lot, like my thoughts or brain was not really functioning well…with the way my thoughts were, if my mother had not been around maybe I would have started having mental illness” [Chipo, female, aged 18] “I didn’t see other people because I was worried that I would infect other people…I would just stay on my own because I was afraid I would spread TB.” [Maidei, female, age 22]	“Yeah, in adolescents, nowadays, they are probably… abusing drugs. Would be smoking, drinking a lot of alcohol. So if somebody is taking those things, adherence will be poor.” [#3, female] “The home situation is not very supportive…I am sure they may be coming from poor backgrounds. So [they] will say ‘yeah sister, I am taking my medication but there is not much food at home’” [#7, female] “They will be depressed. But you can notice that as time goes on, they will start to improve in how they talk to you and how they respond to medication” [#5, female] “They don’t take it quite well—you know they think TB, they think they are going to die. They will be depressed.” [#5, female]		“[AYA] are abusing…it’s like a mild opioid that you take for cough…And yeah, so I mean that’s a huge challenge and underreported” [#4, female, regional] “I think you need to address the [personal] stigma aspect so that they can feel comfortable to present whenever they have signs and symptoms of TB.” [#1, male, national] “The outcomes really are worse off in cases where you don’t have that support, that clear support from the family” [#7, male, regional] “Some are stressed, some end up being psychotic or depressed…I think these are really issues that may not have been prioritized strongly by the country. There is a gap there.” [#3, male, national] “There’s not a specific mental health program for those with TB, even though of course, we know that they have more depression.” [#4, female, regional]
**Domain**			**Family/Community**	
**External stigma**	“Well with my husband the relationship deteriorated because he was acting like am lesser than him that’s why I got infected with TB and he would laugh and look down at me.” [Petronella, female, aged 17] “Yes, I feel very discriminated upon because they shouldn’t be saying I can’t come in [to store] because I have TB” [Stembile, female, aged 19] “Yeah [I have felt discriminated]. There are times when even saying good morning to someone feels like they think you have infected them. Even if they reply I can tell that they really did not want to so I end up not saying anything at all sometimes.” [Chidochase, female, aged 19] “Especially the people at my school, I really felt neglected, like when you would go and sit on a bench, no one wanted to talk to you, no one wanted to be where you are, no one.” [Chipo, female, aged 18]	“Those who are struggling—some fear stigma. They dont want to be seen by society [and] coming to the clinic. People want to know why, every time, every week he goes to the clinic. Its a problem. So some end up not coming to collect their drugs… its a problem” [#7, female] “TB is discriminated, yes. But it is most sensitive in this age-group. If it starts with the adolescence, the early puberty, up to say 24 years—They are in the sensitive years like that is the age group where they start dating, start knowing about their image, they start knowing about their identity and stuff like that. So if [that] is messed up with TB infection–it doesn’t go down well.” [#6, female] “The 10–15 is easier to take treatment, but the 15–19 they have a lot of stigma and discrimination around them, they feel the community may disown them when they are sick.” [#8, female]		"Stigma is a big challenge in Zimbabwe. I think particularly in that age group of adolescence—it can be a serious issue especially for those who are still in schools” [#2, male, national “So stigma [in communities] is serious, actually—and needs to be [addressed]—its an issue particularly for the adolescents.” [#2, male, national]]
**Inadequate treatment support**	“They need to be given encouragement, because when you see someone opt to not take the medication **i**t means there is something wrong—its either they are not getting the support they need or have someone to keep encouraging them to keep taking the medication” [Mukudzei, male, aged 23]	“If they are orphans, they are moved from one guardian to the other—and it’s a challenge with support… this contributes to their treatment success or failure” [#9, female] "Usually the ones from a poor family—they will not be having a good caregiver, like no one will be watching them or asking them if they have taken [their] tablets. If you dont have someone on your side saying do this and do that, they don’t respond as well [to adhering to medication]” [#5, female]		“So definitely they need that support at home. So the outcomes really are worse off in cases where you don’t have that support, that clear support from the family” [#7, male, regional]
**Domain**			**Provider/Health Facility**	
**Youth-unfriendly services**	“As they do not work on weekends it becomes hard as a student because I might not be able to balance out collecting on a day during the week.” [Themba, Male, aged 23] “We should also get counseling and provided with proper information and not just bombarded with pills.” [Chidochashe, female, aged 24] “Although the doctors are the ones who are supposed to handle the people with TB nicely, the way they are rough with people and the way they act or behave, it just shows they do not have any care for people with TB” [Mukundi, male, aged 24] “Because it reduces someone’s dignity when they are being shouted at and exposing your status in-front of people” [Chipo, female, aged 18] On that bench we sit, there are a lot of people sitting there, who have come for different reasons, so now when they give me my tablets there, someone does not know what the tablets are for, they can actually start thinking I have AIDS. So I just expect privacy, where I am taken somewhere privately to give me my pills” [Mukudzei, male, aged 23]	“Some of them are school goers, so I think that one problem when [they write] exams. So sometimes it will clash—you end up losing that patient for that day.” [#2, female] “You give them their tablets, maybe give them health education if it’s their first time. But sometimes you dont have time because of a shortage of staff.” [#4, female] “…because the adolescents–they value their privacy too much.” [#8, female] “I think he did not get like full like proper health education—he thought maybe I finish the two months I am healed.” [#5, female] "With adults, we won’t be talking about cosmetics—but with the adolescents you have to talk about the cosmetics part of it. Because isoniazid—it causes skin rash. So if that appears and you didn’t sensitize them to that, they will stop taking the medication. But with adults, even if you tell them or don’t tell them, they’re not worried about their beauty.” [#6, female]		“The challenge is [having] alternative timing like organizing the timing of medical appointments to fit into their lifestyle.” [#9, female, international] “The more ideal model is what you have in resource-rich settings…where we have TB program nurses who can provide treatment support…and will support that family, the whole way through.” [#8, male, international] “One of the issues that needs to be strengthened is the capacity of the healthcare workers to work with adolescents. They are more used to dealing with children and adults so they are not really friendly to adolescents” [#1, male, national] “Most young people they value their privacy, they would want to get treatment without–maybe if one is below 18 or 16, they require parental consent–some of them they may not be able to get that because they value their privacy. [#5, male, regional]
**Resource limited clinics**	“The clinics we would go to they would say they do not have the resources for testing” [Mukundi, male, aged 24]	“The main challenge at this clinic is that of understaffing. We are severely understaffed—so much that—we are supposed to be 20 and right now we are only 5. So its very difficult.” [#9, female] “But when it comes to drug shortages, this is also a challenge. When it comes to transport—we cannot have a fund to give them.” [#3, female] “TB drugs are most of the time out of stock. So if its out of stock—it means in all of Zimbabwe its out of stock” [#6, female]		“We are so resource limited. It’s like when they get ill there is so little support they can get” [#4, female, regional] “With the issues of access to diagnostic services…in the rural areas, it could be resource challenges.” [#3, male, national]
**Domain**			**Policy/Health System**	
**Absence of AYA specific data and guidelines**		“We don’t have data for those age years—I don’t think” [#5, female] “I think there is need for further training [in AYA care] and guidelines” [#9, female] “Yeah I think as they grow older there is some sense of responsibility—unlike the 10–14 they need someone like a caregiver. But we need to have better guidance [on AYA care]” [#9, female]		“The age-disaggregated data and I think the mortality data is the one that we need to advocate that there’s a higher need for adolescents and young people.” [#4, female, regional] “Yeah in Zimbabwe we got the national TB guidelines…but in terms of adolescents having specific policy on adolescents themselves, we do not have that one.” [#1, male, national]
**Paucity of TB awareness**	“I am usually around or associated with people that smoke marijuana. So, I think when we are sitting together and they are smoking maybe that’s where I got infected [because I think you get TB from smoking]“[Chipo, female, aged 18] “I was seriously sick and people could have thought it was spiritual attacks because when we went to the clinic they would detect nothing.” [Mavis, female, aged 22]	“I think there are misconceptions of the community, that they correlate TB with HIV. That’s a very big challenge” [#9, female] "There was a young guy and he asked me ’hey sister I heard that this tablet will cause me to have a low sperm count’” [#6, female]		“I think one of the issues of course, issues of health education and health promotion—in some of those areas which are very remote in the country.” [#7, male, regional]

Mukudzei is a young man aged 23 years, who had encountered multiple delays in being diagnosed with TB, including through a series of misdiagnoses (*facility and policy)*. By the time that he was correctly diagnosed with TB he was very ill. He reported receiving very limited counseling upon his diagnosis and initiation of treatment, and with little previous TB knowledge to draw on, his misunderstandings about TB persisted. He presumed that TB was fatal and in the absence of accurate and reassuring information he became more fearful, unaware of the curative nature of the treatment that he was taking *(facility and policy)*. Although his close family and friends supported him to broadly adhere to treatment, he described feeling extremely isolated and lonely as he was discriminated against by individuals in the community who were not part of his close circle *(community)* and he further chose to self-isolate. Dispirited by initial reactions within his wider social circle, he chose to conceal his TB status. The side effects of the TB treatment impacted his energy levels and subsequent employability as he was unable to start a new job. The related precarity of not being able to work and generate an income for himself exacerbated his anxiety and sadness about his illness *(individual)*.

A similar story was shared by Tonderai, a young woman aged 19 years, who experienced a two-year delay before receiving a diagnosis. She described experiencing hostile care at the multiple clinics she visited before eventually receiving a confirmatory TB diagnosis *(facilit*y *and policy)*. The protracted amount of time between the onset of symptoms and being diagnosed meant that she had already missed a significant amount of school before commencing TB treatment *(facility and policy)* and the related out-of-pocket healthcare costs had put cumulative economic pressure on her household *(individual and community)*. Her limited school attendance meant that she missed her school examinations, disrupting her educational progress and pushing her back behind her peers. She described feeling very isolated, shunned within her family and by her community because of her illness *(community)*. She felt as though she was the only young person with a TB diagnosis *(individual)*. Although she engaged with treatment, her limited TB literacy exacerbated both her anxiety about her prognosis and her isolation which in turn further undermined her engagement in care and treatment.

These two case studies have broadly similar features, phases and common challenges that were experienced by AYA in this study in managing TB that emerged across all three datasets (AYAs, healthcare providers and policy stakeholders). These experiences highlight protracted delays to TB diagnosis, the compounding consequences of limited investment in TB literacy and an absence of tailored psychosocial support. Without attention to the specific needs of AYA, the physical effects of TB and its related treatment disrupted critical AYA social and educational engagement, as well as their employability and personal or household income.

Within the AYA sample there was one exception. Maidei, a young woman aged 22, had initially encountered financial and educational challenges similar to the two case-studies above, but the consequences of these were mitigated by the support and guidance provided by her mother, who herself had previously had TB. Unlike the other AYA, Maidei could benefit from the relatively high pre-existing TB literacy within her household to help her understand her prognosis and manage her expectations of the fluctuating side-effects of treatment. This enabled her to approach her TB treatment journey with less fear. Knowing that the treatment was curative and receiving support from her family, she felt motivated to adhere to treatment. Aware of her mother’s earlier diagnosis and recovery, she described feeling comforted that she was not alone and that her current discomfort was temporary and that she too could get better.

In the interviews with healthcare providers and policymakers, similar concerns were echoed. They described delays in diagnosis, limited knowledge of TB in the community, self-imposed isolation, a paucity of counseling and health information for AYA at the start of treatment and the unfriendliness of health services to adolescents with TB. The common features of AYA experiences are organised by the four socio-ecological domains below in Table 1.

### Proposed interventions

In response to the needs of AYA with TB, participants proposed interventions to address these shortcomings (further highlighted in Table 2 below). While interventions may be implemented at a policy level, their influence is likely to percolate down through the socio-ecological domains, precipitating into health facilities, communities, and individuals. Policy level interventions are described first, recognising that they will exert an influence on all other domains. Individual interventions are omitted as they are largely resolved by policy, facility and community level interventions.

**Table 2:** Study participant recommended interventions to address identified AYA TB care challenges.

Major Theme	Specific Challenges	Possible Interventions
**Individual**		
Financial issues	• Transport costs • Insufficient nutrition	• Social protection government subsidies (i.e. food, cash, multi-sectoral)
Disrupted education	• Inability to attend school • Delayed life opportunities	• Adapting AYA clinic visit times • Shortening isolation requirements
Psychosocial challenges	• Limited knowledge of TB • TB stigma and fear • Mental heath challenges (i.e. depression, anxiety, loneliness)	• TB education • Intensive counseling • Access to mental health services
**Family/Community**		
External stigma	• Discrimination in community	• Community-led TB awareness
Inadequate treatment support	• Lack of adherence support	• Other sources of adherence support (i.e. technology, peer groups)
**Provider/Health Facility**		
Youth unfriendly services	• Concerns of confidentiality • Unfriendly staff	• Reassuring delivery of confidential care • Training healthcare providers in AYA-specific TB care
Resource limited clinics	• Lack of resources/staff	• Prioritization and policy changes for AYA TB care including resource allocation
**Policy/Health System**		
Absence of AYA specific data and guidelines	• Lacking AYA TB data • Insufficient guidance on AYA TB care	• Mandate age-disaggregated data • Develop AYA specific TB guidelines, toolkits and national plans
Paucity of TB awareness	• Lack of TB awareness	• National stigma-reduction and awareness campaigns

### Policy interventions

To gain attention and allocate appropriate resources towards AYA TB care, health care providers and policymakers recognized that it was necessary to develop policies that state the need for youth-centered services. As one policymaker (*#3*, *male*, *international*) argues:

“*[if we] actually mainstream them as a focus in those policy guidelines or policy documents, then we know that everyone in the country is going to focus upon them. So, we need that as number one, the policy guidance and their inclusion within policy*.”

There was also an appreciation of the value of global leadership and advocacy in triggering youth-centred policy shifts at a national level and to facilitating the development of operational guidelines. There was consensus that AYA would be more readily prioritized if age-disaggregated data were available showing the burden of TB amongst this age-group. Policy makers emphasized the value of WHO recommendations to collect age-disaggregated data, if such data are analyzed as part of the Global TB report, they would be constructive in catalyzing the implementation of youth-centered policy at a national level.

Healthcare providers described the need for further guidance to reduce their clinical uncertainty about how to adapt treatment and care practices to AYA to better respond to their specific needs and challenges. For example, participants described the need for greater international leadership in advocating for reducing the duration of mandatory isolation that is recommended for AYA initiating TB treatment. This in turn would help the National TB Programme ensure that AYA are not required to isolate for any longer than is clinically necessary. Redressing the harms of unnecessarily prolonged isolation could alleviate many of the consequent psychosocial challenges AYA experience including inhibited social lives and disrupted educational and income-generating opportunities.

To further ameliorate the social harms of being associated with TB, all three participant groups highlighted the need for more investment in nationally implemented TB education and stigma-reduction campaigns. They argued that these should be focused on the general population, including communicating that AYA may be affected but that treatment would eliminate transmissibility and was curative.

All three groups recognised that social protection was required to mitigate the catastrophic financial impacts incurred by households, and individuals, when supporting AYA through TB treatment as a mechanism to cushion against the negative socioeconomic consequences of TB. Health care providers also highlighted the need for subsidized transport support, incorporated into the government funding of TB care, to ensure AYA were able to attend clinic appointments.

### Facility level interventions

All stakeholders agreed that additional training should be provided to healthcare providers working at facilities providing TB care to AYA to increase their awareness of the high TB risk among AYA and to provide youth-friendly and confidential care. This was strongly recommended by AYA themselves, who described how being confident that their healthcare providers knew how to engage with young people and “*actually knows who you are and sits down with you and starts talking with you*.*” (Mukudzei male*, *aged 23)* would elevate their trust in the care they receive and support engagement. This was reinforced by healthcare providers themselves, some of whom spoke about their lack of confidence in their ability to provide effective, youth-appropriate care:

*“This is the most difficult group to deal with—the adolescents—so I think the training should be ongoing*. *There should be refresher courses so that we keep in line with their needs” (healthcare provider #9*, *female)*.

**Fig 1:**
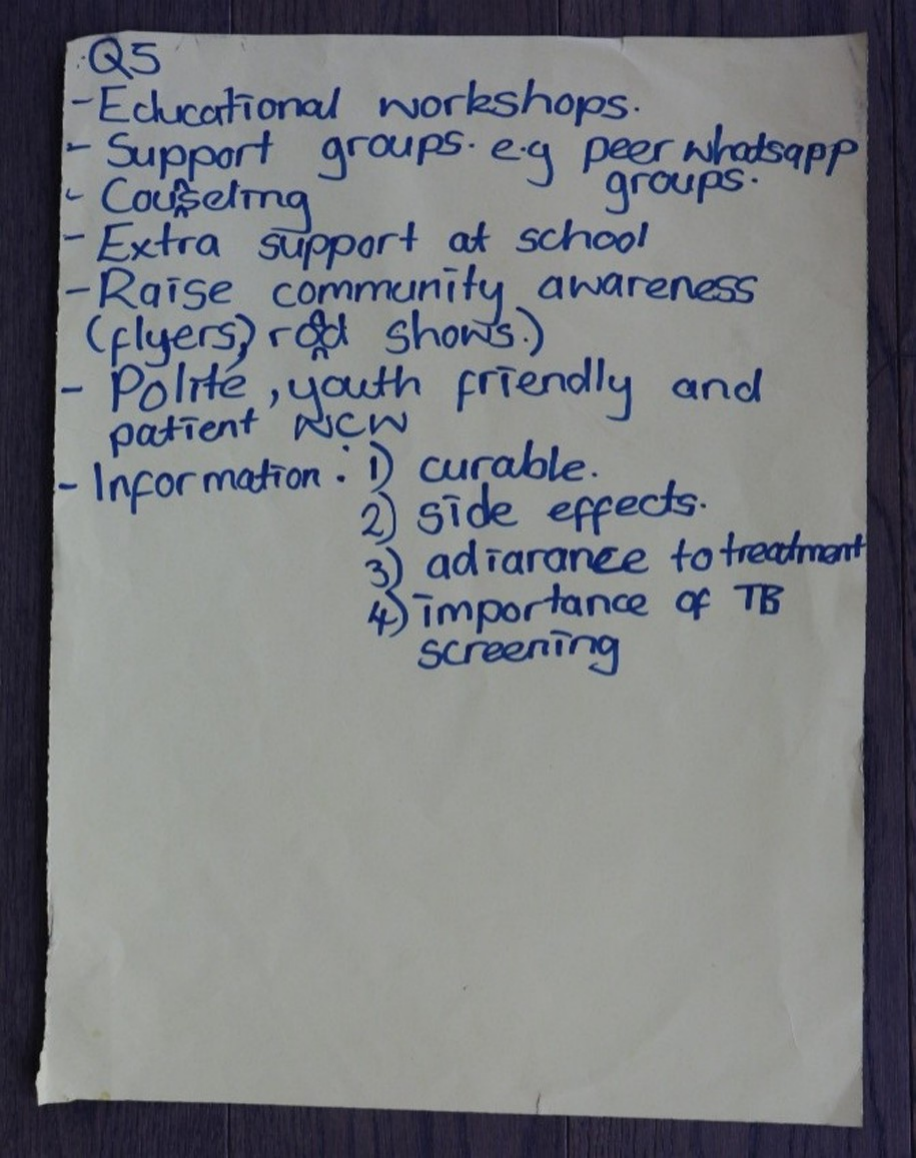
Brainstorm of AYA friendly services during participatory workshops.

There was also consensus about the need for youth-centered models of care. Four core components were identified that should underpin youth-friendly TB services: 1) adapted clinic-opening hours to fit around school or work hours; 2) provision of youth-friendly spaces where AYA could meet their peers affected by TB; 3) access to and ongoing counseling to support TB literacy; 4) delivery of differentiated psychosocial support care, potentially provided by peers, to encourage sustained adherence and to improve mental health. Examples of these youth-friendly services were brainstormed and captured by AYA during the participatory workshops as seen in Fig 1.

The value of provider-led counselling and psychosocial support was widely recognized, but particularly emphasized by AYA who considered it as vital for optimal adherence. As one AYA (*Sibusisiwe*, *female*, *aged 19*) shared:

“*They should have counsellors that tell adolescents that if you take your pills without skipping, TB will be treated in the stipulated time frame that you are given. But if you take the pills and you skip some days, which will affect you in the future.”*

There was also enthusiasm across all three groups for investing in the development of peer support groups that could further extend AYA-tailored TB education and stigma reduction, as well as redressing the widespread isolation experienced by AYA. To ensure that AYA can be connected to peer support soon after their TB diagnosis, participants proposed digital platforms for engaging with support even when needing to be physically distanced from others: “*Peer support is something we can learn from HIV… we can use technologies or meet outside or join the group once you’re not infectious…but there are ways to do it*” (*policy maker #9*, *female*, *international*)

### Interventions at community level

Implementing specific local interventions that mitigate the impacts of stigma, mental health disorders and limited social support within communities was stressed by AYA, healthcare providers and policymakers. Stigma was recognized as an important barrier to AYA engagement in care. Community-led campaigns to increase public awareness about AYA’ susceptibility to TB, TB symptoms and ways to access TB testing if warranted were suggested as a possible solution.

Engaging AYA in the development of community-based programmes was considered critical to the to the success of such campaigns: “*You also need to hear the voices of these adolescents*, *in terms of their experiences*, *what they would want*, *how they want the services to be provided and who they want to provide these services to them*.” (*Policy #7*, *male*, *international)*.

## Discussion

TB among AYA is a global concern, yet our study supports other emerging evidence to demonstrate that existing services do not meet their needs [33, 34]. This qualitative study triangulates the perspectives of those involved in AYA TB care, including AYA themselves, healthcare providers and policy makers. These three groups represent considerable expertise, informed by lived experience and professional practice, and it is vital that we incorporate their knowledge in framing the needs of AYA and devising solutions that are going to work *for them*. Given the social determinants, developmental concerns, impact on livelihoods and life trajectories of AYA in TB care, the socio-ecological framework used in this study allows to illuminate the interconnectedness of their challenges across the domains and highlight intervention opportunities which could exert positive influences across multiple levels shaping AYA TB care [35, 36].

The experiences of AYA undergoing TB treatment revealed multiple sources of physical, mental, and social stress encountered during the diagnostic and treatment periods. While TB-specific challenges are also experienced by adults with TB [37–40] including debilitating symptoms, depression, social isolation, impoverishment, and post-TB lung disease, the circumstances in which they are faced are unique to AYA. They are made more acute by having to manage them while simultaneously navigating a period in their lives which is characterized by physical, cognitive, emotional, social, and economic changes [41], which is commonly experienced as challenging for most people regardless of disease status. TB and other diseases add another layer of complexity to this foundational stage of life, exacerbating the demands placed on them and forcing different concerns into conflict with each other. Health systems should not just acknowledge the interacting challenge AYA face but cater for their specific needs given that not doing could have detrimental impact on health outcomes.

AYA undergoing TB treatment described long diagnostic delays lasting up to two years. They highlighted this as the most difficult aspect of their TB journey. Diagnostic delays are experienced by all people affected by TB resulting in increased morbidity, mortality and out-of-pocket costs [4, 5, 42, 43]. However, TB prevalence surveys have shown that the proportion undiagnosed is highest among older adolescents and young adults compared to older adults [44–46]. The lack of awareness about risk of TB in AYA among healthcare providers and the community may result in even longer delays among AYA, highlighting the value of enhanced awareness and training at the primary care level.

Social isolation made AYA undergoing TB treatment feel lonely, sad and depressed. In many settings social isolation is often recommended and imposed by the TB programme as a means to prevent onward transmission. However, we also found that to pre-empt anticipated adverse social reactions, some AYA and their families felt it necessary to extend or impose additional isolation periods. Similar results have been reported from other studies conducted among AYA affected by TB in Peru, Ukraine and Russia [47–50]. TB related stigma is not unique to AYA and is also experienced by adults [51–53]. However, given AYA have much lower personal resources, the effects of stigma may be magnified and can negatively affect self-esteem among AYA [54–56]. Alongside this, AYA in this study also consistently described that being diagnosed with TB profoundly impacted their ability to actively engage in schooling, employment, and general life which were all considered to impede their potential achievements. Guidance on how long AYA with TB should stay away from school is limited. National TB guidelines often do not address requirements for isolation, those that do such as in Peru, often mention the duration of self-isolation and exclusion from school as recommended a minimum of two months [33, 50, 57]. Quantitative data on educational attainment and school disruptions among AYA affected with TB is generally lacking. However, given outdated existing guidance in most countries it is likely that AYA with TB miss several months of school resulting in delayed graduation and school dropouts fueling a cycle of intergenerational poverty.

Our findings reflect that there are commonalities in the realities of experiencing a significant life-threatening condition, such as financial constraints and resource limited clinics, which are shared by both adults and AYA engaged in TB treatment and care. However, this study identifies specific conditions which pertain to AYA and which shape the particularities of the challenges that they face and the interventions that are needed to address them. For example, although the clinics that TB patients attend in Zimbabwe are often overstretched, the limited resources available mean that rationed care tends to be orientated towards the needs of adults, rather than AYA. This perpetuates a lack of attention to recognizing and meeting adolescents’ needs, further embedding adolescent unfriendly services and limited investment in training for HCWs. In addition, our study identifies AYA specific needs, for example disrupted education and how this adversely interacts with AYA’s social relationships. This influences the psychosocial challenges that they face within the specific developmental stage of adolescence. While mental health is likely to impact treatment engagement for all those in TB care, the particular challenges that AYA experience requires tailored responses to their social conditions and the priorities of this age group.

The interventions recommended by AYA undergoing TB treatment, healthcare providers and policy makers in this study have a strong similarity with those recently laid out in a consensus statement [58] from an international expert panel namely reporting age-disaggregated data, healthcare provider training in AYA care, youth-friendly TB services and clinics, and community-based models of care. While youth-friendly TB services and peer support were key interventions recommended by the expert panel and the stakeholders participating in this study the “how” (i.e. design and implementation) so that it can fit specific contexts, including health systems, is yet to be decided. Successful youth-friendly models of care and peer support for HIV [59], sexual and reproductive health [60], and other chronic health conditions [61], can serve as a guide for developing and implementing youth-friendly TB services, including peer support, extending services to after-school hours, and counseling [62]. Importantly such services should be developed together with AYA [63], as was noted in this study.

Our research has some important limitations. Due to the qualitative nature of the study and relatively small sample size, we were unable to explore how AYA’s experiences vary by socioeconomic status, HIV status or caregiver support. Notably, the triangulation of perspectives in the care network surrounding AYA affected with TB excluded caregivers, other family members and friends. Their views would have provided informative insights into what structures and support could or should be available at home. Similarly, the use of purposive sampling comes with important limitations. Our sample of AYA participants were mostly adherent which likely is not generalizable to the AYA population and further research is needed to consider non-adherent AYAs. Despite these limitations, our study employs the perspectives of AYA with TB, their healthcare providers, and policymakers to provide an insightful assessment of the healthcare needs and challenges of this key population. The results of this study will hopefully inform intervention development and policy considerations.

In summary, optimizing engagement in TB care for vulnerable AYA with currently unmet needs urgently requires interventions to mitigate the impacts of stigma, poverty, mental health challenges, and adherence. Programmatic and policy changes are warranted at all levels of the socio-ecological framework including individual, community, provider, and health system. Further research is necessary to investigate the effects of proposed interventions and implementation at scale.

## Supporting information

S1 File Interview guides. Workshop Tool for Patient Participants > 16years old. Interview Questions for Patient Participants. >16years old. Interview Questions for Healthcare Worker Participants. Interview Questions for TB Policy Stakeholders.
(DOCX)

S1 Checklist (DOCX)
