# Supplementary material for: Person-centered and youth-oriented interventions to improve TB Care for adolescents and young adults
Source: PLOS Glob Public Health. 2024 Nov 15;4(11):e0003659. doi: 10.1371/journal.pgph.0003659 (PMC11567634; doi:10.1371/journal.pgph.0003659)
Supplement: S1 File — Workshop Tool for Patient Participants > 16years old. Interview Questions for Patient Participants. >16years old. Interview Questions for Healthcare Worker Participants. Interview Questions for TB Policy Stakeholders. (DOCX) [file pgph.0003659.s001.docx]

Contents

[Workshop Tool for Patient Participants >16 years old 1](#_Toc180337112)

[Interview Questions for Patient Participants >16 years old 4](#_Toc180337113)

[Interview Questions for Healthcare Worker Participants 7](#_Toc180337114)

[Interview Questions for TB Policy Stakeholders 9](#_Toc180337115)

# Workshop Tool for Patient Participants >16 years old

*Exploring the psychosocial impact of tuberculosis in adolescents and gaps in service provision: a mixed methods study in Zimbabwe*

**Introduction**

Thank you for taking the time to join our workshop today.

My name is ____________ and I am working with BRTI on the research project looking at how adolescents and young people experience TB diagnosis and treatment. The purpose of the study is to know how to improve care for adolescents who may have TB in the future through high-quality, youth-friendly health services in Zimbabwe. You have been selected because you are an adolescent living in the community where our research will take place. I would like talk to you about your experiences of **obtaining healthcare and support for TB**. I also want to ask you about **how TB affects your life** including your mental state, your day-to-day activities such as school and work and your relationships with friends, family and peers. I am also interested in **suggestions you have for what might help adolescent’s access health services and how to ensure that the services meet their needs**. This workshop will be 4 hrs long with a 45min lunch break. We value your opinion and appreciate your willingness to contribute your time out of a busy schedule.

I would like to record the discussion in order to make sure that I capture all of the valuable information. We will also write things down while we’re talking so that we don’t forget anything. Everything said at this meeting is confidential. If you have any questions about this study, you can ask me now, or at any time during the workshop.

| **Session** | **Specific Topics/activities** |
| --- | --- |
| **Introduction Session: Ice breaker and Introduction**  *Getting to know participants and building rapport, trust, and engagement* | **10:00-10:45 (45 mins)**  **1. Welcome and introductions of facilitators:** Welcome the group members and introduce yourselves, and how you came to be leading the group.  2. **Rhyming names**: Go around the group and allow everyone to introduce themselves with a ‘rhyming’ adjective (i.e. starting with the same letter) for example Smiling Shayla, Talkative Tessa, Crazy Christine.  3. **Introductions through** **Buzz pairs:** Ask attendees to turn to the person on their left to tell each other 1. their name, 2. one thing they like doing, and 3. what made them interested in coming to join the session today. Then get up form new pairs and share same information and one fun fact about themselves. Repeat process once more.  4. **Close Activity** by thanking everyone and encouraging everyone to try and mingle and get to know each other as much as possible over the course of the workshop and during the tea-break. |
| **Session 1: Impact of TB Diagnosis on Life – mental health, work/school, relationships**  *We would like to understand how TB has impacted areas of a young person’s life i.e. their mental health, school/work/other activities and their relationships with friends, family and peers. Has being unwell resulted in other concerns i.e. financial difficulties, etc.* | **10:45-11:40 (55 mins)**   1. **Set up:** Ask participants to sit around a table, on which has been laid out a piece of A3 coloured card per participants, plus pens, scissors, magazines, glue. 2. **Explaining exercise:** The facilitator explains that the exercise is to create a collage of their own experiences of getting a TB diagnosis and how it has impacted other areas of their life. This could be through drawing of who was with them when they got diagnosed, who helps them adhere to medication, if they have had to pause going to school or work, or including images representing the challenges they face with TB. If participants do not feel at ease with their ability to draw, they are encouraged to include writing and words which might help them express themselves.   The facilitator could provide an example of a collage on card, including for example, a hand drawn figure of two individuals – one representing the adolescent patient and their grandmother (to represent them going to the health clinic together), a picture of school with a cross through it (to represent not being able to attend school), and a picture of someone looking sad (to represent their mood/mental health). Explain that this is an example of visually mapping their experience, but that they can choose whatever images/ drawings they feel represent their experience with TB.  Explain that some of the things we are interested in understanding are how a TB diagnosis changed their day to day activities, their mental mood, and their relationships.   1. **Creating collages:** Give participants around 30 minutes to create their visual collages. Give them a 5-minute warning before the time is up. Stick up all the collages on the wall with blue tac. 2. **Presenting collages:** Ask each participant in turn to explain their collage and what the different pieces represent. Ask probing questions if the participant doesn’t describe fully, such as what particular pieces on the collage represent, and how it links back to their experiences with having TB as a young person. It will be useful for facilitators to take detailed notes during these presentations for interpretation of collages.   [While adolescents are creating collages]: Go around and ask them to fill out specific characteristics: How many weeks since they were diagnosed with TB? Are they male or female? HIV- and HIV+ adolescents? Married and not married? Are they parents/not parents? |
| **Tea break/ energiser** | **11:40-11:50** |
| **Session 2: How well do existing services meet adolescents’ comprehensive TB care needs?**  *We would like to understand what a young person’s journey with from TB diagnosis and through treatment has been and what challenges do they face on a day-to-day basis* | **11:50-12:35 (45 mins)**  **List and rank: Barriers to engagement with care.**  1. **Facilitator explanation of the session:** Facilitator describes that we are interested in understanding how well existing services meet adolescents’ comprehensive TB needs. We are interested in looking at the following questions   - *Are there any services that are needed for adolescents but which are not provided by this facility? If yes, then why not?* - *How do adolescents currently provide feedback to health facilities about their service needs?* - *Is there a specific day or time in the week when adolescents are encouraged to attend health facilities? Do adolescents have to pay for any services?* - *Is there anything that the health facility does to make seeking TB care easier for adolescents?* - *Do you know what adolescents want in terms of information, services, and how the service providers interact with them?*   3. **Buzz groups:** each group is given pens and multiple pieces of card (around A5 size). Ask each group to discuss and write on their card some of the barriers they face in accessing health care. When they have written them, ask them to stick them up on a wall, so all the groups’ cards are put up together, and try to group ones that are similar together.  4. **Circle:** Ask all participants to come back together as a full group. Ask one person from each group to present to everyone the barriers they wrote. If some of the cards repeat the same barrier, then stick them on top of each other on the wall, so each barrier is shown once.  5. **Diamond** **Ranking**: Ask the group to arrange the barriers in a diamond shape with biggest or most significant barrier at the top and going down in significance with a diamond shape.  6. **Discussion**: Discussion as a whole group about which barriers participants ranked as the most important. |
| **Lunch** | **12:35-1:20 (45 mins)** |
| **Session 3 : How well do existing services meet adolescent’s TB needs ? Ideas for youth-friendly support during TB service provision**  *We would like to understand TB service provision from a young person’s perspective how they would prefer to be supported.* | **1:20-1:50 (30 mins)**  **What would your ideal support scenario be?**  **1. Facilitator explanation of the session**: Facilitator explains the purpose of the session that we would like to understand what their ideal support scenario would be for their TB care. We would like to hear from them what they think would best support them.  2. **Buzz groups**: Facilitator asks participants to talk to the person next to them about what they would most want for support or their health.  3. **Group discussion**: after a few minutes of buzz groups, the facilitator asks for one point from each pair to kick off a whole group discussion, and then allows others to contribute further points. The facilitator writes points up on a flipchart. |
| **Closing and transport refund** | **1:50-2:00 ((10mins)**  Facilitator thanks the group for sharing, and reiterate that the point of the workshop is to hear from them about their experiences and views.  **Closing game ‘Message to you’**  **Stick cars on back:** Ask the group to stand in circle facing the back of the person in front of them. Give everyone a sheet of card and a marker, and ask them to write their name at the top of the card. Now ask them to hand it to the person behind them who will stick it on their back with masking tape.  **Writing messages**: Ask group members to mingle and write an appreciation on each person’s card, to acknowledge what they have brought to the group. Alternatively, ask group members to draw faces showing how they feel about that person.  **End the session** with one round of everyone: How did you feel about today? One thing you will take away from being part of this research?  Give out transport refund to each participant. |

# Interview Questions for Patient Participants >16 years old

**Exploring the psychosocial impact of tuberculosis in adolescents and gaps in service provision: a mixed methods study in Zimbabwe**

Introduction

This introduction aims to:

1. Explain the purpose of the interview.

2. Emphasize that the interviewees are the experts and for which we would like to learn their experiences and perspectives.

3. Ensure confidentiality. We will not share what you tell us with anyone, including parents / guardians, the health centre, etc.

4. Explain the recording and the importance of speaking loudly.

‘Good morning. I have come to talk with you since you are a young person who has received attention for tuberculosis and I want to know the experience you have had in this situation. The purpose of this interview is to learn from what you told me, to know how to improve care for adolescents who may have TB in the future. It is important that you know that this is a conversation, it is not a questionnaire, and therefore there are no incorrect answers, everything related to the illness you have faced is important to me. To be able to remember everything we talk about, I'm going to record our audio conversation with a small recorder that will capture only our voices, this is because I could not take note of everything we are going to talk about, this audio is going to be transcribed so we can read it and make an analysis with other interviews, if at any time your name is mentioned at the time of transcribing we will change your name to any other, so there is no way to identify you, after that we will delete the audio file. If there is a question that you do not want to answer, you can tell me and we may move on to the next question, do not feel you have to answer me if you do not want to. Feel free to tell me your experiences whether they are pleasant or not, I will not comment on what we talk with your parents or with the health centre.

So if you agree to do the interview we could start (start recording)’

Today is ........................... and we are going to start the interview by asking you

• How old are you?

- What area does your home belong to?

• Who do you live with?

• Do you currently attend school/college/or work?

**Beliefs/Knowledge About Diagnosis**

1. What do you think was the cause for your illness?

Prompts:

- What do you know about TB and how people become ill with it?
- When and where were you infected possibly?
- Did you have any contact with a family member or friend with TB?
- When you knew that person had TB, how did you react? Did you go to a health facility to be tested?

1. Can you talk about what happened when you were diagnosed and how it affected you?

Prompts:

• How did you find out you had TB?

• What were your symptoms? /What made you go to a health centre?

• How long did it take for your diagnosis?

• Was anyone with you during this time?

- How did you feel about the diagnosis process?

**TB Treatment**

1. Would you please describe your treatment and how it affected you?

Prompts:

- Could you describe a typical day when you were going to take your treatment? (What time did you go, before or after school, how did you go and long did it take, with whom did you go, what did you take the pills with?)
- Who was responsible for making sure you took your medicines every day? You or someone else?
- Did you miss classes/work/other activities to go and take your medicines?
- Did you miss days of treatment? Why?
- Did anything make it difficult to get treatment for TB? (e.g. transportation/ cost)
- Did anyone support you in taking your treatment? (e.g. accompany you, provide transportation etc?)
- Did you have any difficulties with the medicines (e.g. discomfort with pills, side effects)
- Have you ever considered not taking your treatment and why?
- What things are helping you with your treatment? (support from staff/family/change in treatment etc)

**Impact on Life**

1. How are you getting on since your illness?

Prompts:

1. Mood/Mental health/Emotional state

- How would you describe your mood/emotions before getting sick?
- After getting sick, was there a change in your mood? (e.g. with diagnosis or treatment)
- Have you sought any help or spoken to anyone about your feelings?

1. Experience of school/college/job

- How has TB affected your life?
- How has it affected your experience of school/college/work?
- Has TB brought any financial difficulties?
- Has being unwell brought you any problems? How did you deal with them?
- What are you concerned about most?

1. Relationships/Social interactions

- In general, how is your relationship with your family? How is the relationship with your parents before and after having TB?
- How was your relationship with friends/partners before and after having TB?
- Has having TB affected your sexual experiences? How?
- Who did you tell you had TB? Tell me why you decided to tell that you had TB to that person or people?
- Have you ever felt discriminated against because you had TB?
- Did you have support from anyone and how did they help you?

**Medical Care**

1. How have you found the care you have received for TB?

Prompts:

- How satisfied are you with the TB care you received at the health centre/hospital?
- How do you consider the treatment provided by the health personnel has been? Did you ever have a situation that caused you discomfort or receive bad treatment?
- What information did the health staff give you about TB and TB treatment?
- Who from or how do you get information on TB or how would you prefer to get information?
- If you could improve care for adolescents with TB, what changes would you make?

a. Regarding the diagnostic process

b. Regarding the treatment process

- What do you think could help teens learn about the importance of being evaluated if they have had contact with someone with TB, and the importance of taking all the medicine properly?

**Conclusion**

1. What part of this experience of getting sick with TB has been the most difficult?
2. What has helped you most through this experience?
3. Is there anything else related to your TB experience that you would like to tell me about or that is important for me to know?

Thank you for your time, do you have any questions for me?

# Interview Questions for Healthcare Worker Participants

**Exploring the psychosocial impact of tuberculosis in adolescents and gaps in service provision: a mixed methods study in Zimbabwe**

**Introduction**

This introduction aims to:

1. Explain the purpose of the interview

2. Emphasize that the interviewees are the experts and for which we would like to learn their experiences and perspectives

3. Ensure confidentiality.

4. Explain the recording and the importance of speaking loudly

Good morning, I have come to talk with you since you work in a health centre where you provide care for adolescents with TB and the purpose of this interview is to learn from what you tell me, to know how to improve care for adolescents who may have TB in the future. It is important that you know that this is a conversation, it is not a questionnaire, and therefore there are no incorrect answers, everything related to the care of adolescents affected by TB is important to me. To be able to remember everything we talk about, I'm going to record our audio conversation with a small recorder that will capture only our voices, this is because I could not take note of everything we are going to talk about, this audio is going to be transcribed so we can read it and make an analysis with other interviews, if at any time your name is mentioned at the time of transcribing we will change your name to any other, so there is no way to identify it, after that we will delete the audio file. If there is a question that you do not want to answer, you can tell me and we move on to the next question, do not feel obliged to answer me if you do not want to. Feel free to tell me about your experiences, whether they are pleasant or not, I will not comment on your conversation with your boss or colleagues at the health centre or with your patients.

So if you agree to carry out the interview we could start (start recording)

Today is ...........................and we are going to start the interview by asking

• What is your position at the health center?

• How many people do you work with?

• How long have you been working there?

Questions:

**TB Diagnosis and Treatment**

1. Who transmits TB to adolescents? The family members? The friends?
2. What is the process of TB diagnosis for adolescents?
   1. Is TB diagnosis often delayed?
   2. Are adolescents responsible themselves for seeking care? Or do their parents or family members bring them to clinic?
3. What information do you give to teens and their parents or guardians about TB and TB treatment?
4. Tell me about the process of coming to the health center to deliver anti-TB drugs.
   1. How much time does each teenager need?
   2. Do you know what activities teens usually lose when they go to receive treatment? (classes, work, etc.)
   3. Do you know what activities parents usually lose when they go with their teenagers to receive treatment? (classes, work, etc.)
   4. What other barriers are there?
5. If adolescents suffer any side effects from anti-TB drugs, what does the health center staff do to treat them?

**Psychosocial Aspects of Disease**

1. Have you noticed any changes in mood/emotional well-being after adolescents get sick with TB?
2. Do adolescents confide in their difficulties adjusting to TB diagnosis and treatment?
3. What kind of psychological or emotional support do teenagers receive during their TB treatment?

**Medical Care**

1. What are the main differences you see between TB care with children, adolescents and adults?
2. Have you noticed any association between the improvement of symptoms and the abandonment of treatment in adolescents with TB?
3. What differences have you noticed between adolescents with good adherence and adolescents with poor adherence to TB treatment?
   1. Regarding the socioeconomic situations and levels of education of their families
   2. Regarding the support that their parents / guardians give them
   3. Regarding the beliefs that the adolescent and his family have regarding TB
   4. Regarding the severity of TB disease
   5. Regarding the side effects of medicine
   6. Regarding the psychological state of the adolescent
   7. What challenges do you have to identify, diagnose and treat adolescents with TB?

**Conclusion**

1. If I could make treatment for TB easier for adolescents, what changes should I make?
2. Do you have any ideas regarding helping adolescents adjust emotionally and practically to the life impact of being diagnosed with TB?
3. Do you have any other ideas about the adherence of adolescents to the TB treatment they would like to share?

# Interview Questions for TB Policy Stakeholders

**Exploring the psychosocial impact of tuberculosis in adolescents and gaps in service provision: a mixed methods study in Zimbabwe**

Introduction

This introduction aims to:

1. Explain the purpose of the interview.

2. Emphasize that the interviewees are the experts and for which we would like to learn their experiences and perspectives.

3. Ensure confidentiality. We will not share what you tell us with anyone, including parents / guardians, the health centre, etc.

4. Explain the recording and the importance of speaking loudly.

Good morning, I have come to talk with you since you have a vested interest in TB control and the purpose of this interview is to learn from what you tell me, and to know how to improve care for adolescents who have TB. It is important that you know that this is a conversation, it is not a questionnaire, and therefore there are no incorrect answers, everything related to the care of adolescents affected by TB is important to me. To be able to remember everything we talk about, I'm going to record our audio conversation with a small recorder that will capture only our voices, this is because I could not take note of everything we are going to talk about, this audio is going to be transcribed so we can read it and make an analysis with other interviews, if at any time your name is mentioned at the time of transcribing we will change your name to any other, so there is no way to identify it, after that we will delete the audio file. If there is a question that you do not want to answer, you can tell me and we move on to the next question, do not feel obliged to answer me if you do not want to. Feel free to tell me about your experience and knowledge, whether it is pleasant or not, I will not comment on your conversation with your colleagues.

So if you agree to do the interview we could start (start recording)’

Today is ........................... and we are going to start the interview by asking

• What is your position and its relevance to TB policy?

• How many people do you work with?

• How long have you been working there?

Questions:

**TB Diagnosis and Treatment**

1. Are you aware of the increased risk adolescents have for the development of TB?
2. Are there separate national guidelines for TB diagnosis amongst adolescents?
   1. If not, is there interest/willingness in understanding adolescent-specific TB guidelines?
3. Is routine TB incidence and prevalence data age-stratified for adolescents aged 10-24?

**Psychosocial Aspects of Disease**

1. What kind of psychological or emotional support do teenagers receive during their TB treatment?
2. Are you aware of the increased risk of mental health issues related to disease diagnosis?
   1. Are you aware of any other mental health support for other chronic diseases in adolescents?
   2. Would understanding aspects of mental health on issues of adherence and transmissibility be of interest?

**Medical Care**

1. TB in adolescence is frequently infectious and a source of transmission, are there adolescent-specific adherence support?
   1. Would there be interest in exploring this topic further?

**Specific Policies for Adolescents**

1. Are there any specific guidelines for adolescent-friendly TB services?
   1. Do national registries look at adolescent TB outcomes compared with children and adults?
   2. Are adolescents simply grouped into children and/or adult data?
   3. If not already, is there interest to develop specific guidelines for the adolescent age-group?

**Conclusion**

1. What data or evidence is required to make adolescent TB a policy priority?
2. Do you have any ideas of how to develop better guidelines for the adolescent age-group?
